# Predicting corn tiller development in restrictive environments can be achieved to enhance defensive management decision tools for producers

**DOI:** 10.3389/fpls.2023.1223961

**Published:** 2023-08-03

**Authors:** Rachel L. Veenstra, Trevor J. Hefley, Dan Berning, Carlos D. Messina, Lucas A. Haag, P.V. Vara Prasad, Ignacio A. Ciampitti

**Affiliations:** ^1^ Department of Agronomy, Kansas State University, Manhattan, KS, United States; ^2^ Department of Statistics, Kansas State University, Manhattan, KS, United States; ^3^ Corteva Agriscience Agronomy Sciences, Johnston, IA, United States; ^4^ Horticultural Sciences Department, University of Florida, Gainesville, FL, United States; ^5^ Northwest Research-Extension Center, Kansas State University, Colby, KS, United States

**Keywords:** corn (maize), tillering, plant density, crop plasticity, crop morphology

## Abstract

**Introduction:**

While globally appreciated for reliable, intensification-friendly phenotypes, modern corn (*Zea mays* L.) genotypes retain crop plasticity potential. For example, weather and heterogeneous field conditions can overcome phenotype uniformity and facilitate tiller expression. Such plasticity may be of interest in restrictive or otherwise variable environments around the world, where corn production is steadily expanding. No substantial effort has been made in available literature to predict tiller development in field scenarios, which could provide insight on corn plasticity capabilities and drivers. Therefore, the objectives of this investigation are as follows: 1) identify environment, management, or combinations of these factors key to accurately predict tiller density dynamics in corn; and 2) test outof-season prediction accuracy for identified factors.

**Methods:**

Replicated field trials were conducted in 17 diverse site-years in Kansas (United States) during the 2019, 2020, and 2021 seasons. Two modern corn genotypes were evaluated with target plant densities of 25000, 42000, and 60000 plants ha ^-1^. Environmental, phenological, and morphological data were recorded and evaluated with generalized additive models.

**Results:**

Plant density interactions with cumulative growing degree days, photothermal quotient, mean minimum and maximum daily temperatures, cumulative vapor pressure deficit, soil nitrate, and soil phosphorus were identified as important predictive factors of tiller density. Many of these factors had stark non-limiting thresholds. Factors impacting growth rates and photosynthesis (specifically vapor pressure deficit and maximum temperatures) were most sensitive to changes in plant density. Out-of-season prediction errors were seasonally variable, highlighting model limitations due to training datasets.

**Discussion:**

This study demonstrates that tillering is a predictable plasticity mechanism in corn, and therefore could be incorporated into decision tools for restrictive growing regions. While useful for diagnostics, these models are limited in forecast utility and should be coupled with appropriate decision theory and risk assessments for producers in climatically and socioeconomically vulnerable environments.

## 1 Introduction

Corn (*Zea mays* L.) is a key crop in the global food economy, partially due to predictable phenotypes that enable intensive management. For this reason, high plant densities, optimal planting date, and efficient fertility programs are among the key drivers of high-yielding corn (Duvick et al., 2004; Long et al., 2017; Schwalbert et al., 2018). Concurrently, plant uniformity is targeted to an increasing degree by planter technologies improving singulation and seeding depth for timely emergence (Badua et al., 2021). Even when every effort is made to obtain field uniformity, this goal is arguably idealistic. In reality, plant uniformity may be disrupted by plasticity mechanisms which are often masked, but preserved nonetheless, in the corn genome (Moulia et al., 1999). In addition, corn production is steadily expanding into less productive regions around the world, where such intensive management may not be possible due to climate, socioeconomic status, or other barriers.

Crop plasticity is the ability of a crop genotype to express contrasting phenotypes in an array of environmental conditions (Laitinen and Nikoloski, 2019). Broadly, crop plasticity mechanisms include source (carbon capture) and sink (carbon storage and utilization) manipulations (Dingkuhn et al., 2020). Branching is a common plasticity response, for example, increasing both the source (leaf area) and sink (seed set) potential for a crop plant. Although this phenotypic flexibility is beneficial to individual plants, many modern agronomic crop management practices favor intensification and target uniformity (Matesanz and Milla, 2018). These intensively managed, stabilized environments aim to minimize plasticity expression, which may result in both positive and negative outcomes (von Wettberg et al., 2020). Global interest is mounting in crop plasticity mechanisms as breeders anticipate potential impacts of climate change (Arnold et al., 2019; Schneider and Lynch, 2020). Adaptation to rapidly changing weather patterns and sporadic stress events may be facilitated by plasticity mechanisms conserved in modern crop genetics (Nicotra et al., 2010). The true utility of plasticity is uncertain in modern agronomic settings, as the concept remains mostly theoretical and untested under broad-scale field conditions (Brooker et al., 2022). In addition, the benefit of plasticity at the field-level for relatively determinate crops such as corn is less apparent than for crops such as wheat and grain sorghum, which can be intentionally managed for plastic capabilities via tillering.

Tillers are basal branches of grass crop species, appearing early in plant development for annual crops (Kim et al., 2010b), and further growing and developing in perennial species. As with any plasticity trait, genotype is a strong regulator of tiller expression (Doust, 2007; Laitinen and Nikoloski, 2019). With conducive genotypes, tiller development is encouraged by an abundance of resources and therefore may vary significantly on an individual plant basis based on light, nutrient, or water availability – commonly associated with plant density (Lafarge and Hammer, 2002; Markham and Stoltenberg, 2010). Nutrients key to tiller development are phosphorus (P) and nitrogen (N), as deficiency may prevent expression (Thorne and Wood, 1987; Longnecker et al., 1993; Rodriguez et al., 1999). When soil factors are not limiting, early-season weather conditions are key to tiller development. The relationship between temperature and radiation is commonly quantified via the photothermal quotient (PTQ), which has been correlated to vegetative and reproductive crop mechanisms (Angus et al., 1981; Fischer, 1985; Kim et al., 2010b; Kumar et al., 2016). Within species, genotypes vary in tiller fecundity, even for apparently unrestrictive conditions (Kim et al., 2010a). Tillers can be fertile and develop identically to primary shoots, although delayed in development, usually by a set phyllochron interval (Nemoto et al., 1995). This cumulative age discrepancy sets limitations for the number of tillers able to successfully set seed, as abortion typically occurs in reverse appearance order when resources become limiting over the course of the growing season (Thorne and Wood, 1987). For tillers emerging at opportune times and surviving through the season, yield contributions can be significant in well-managed grains (Lafarge and Hammer, 2002; Pasuquin et al., 2008).

**Figure 1:**
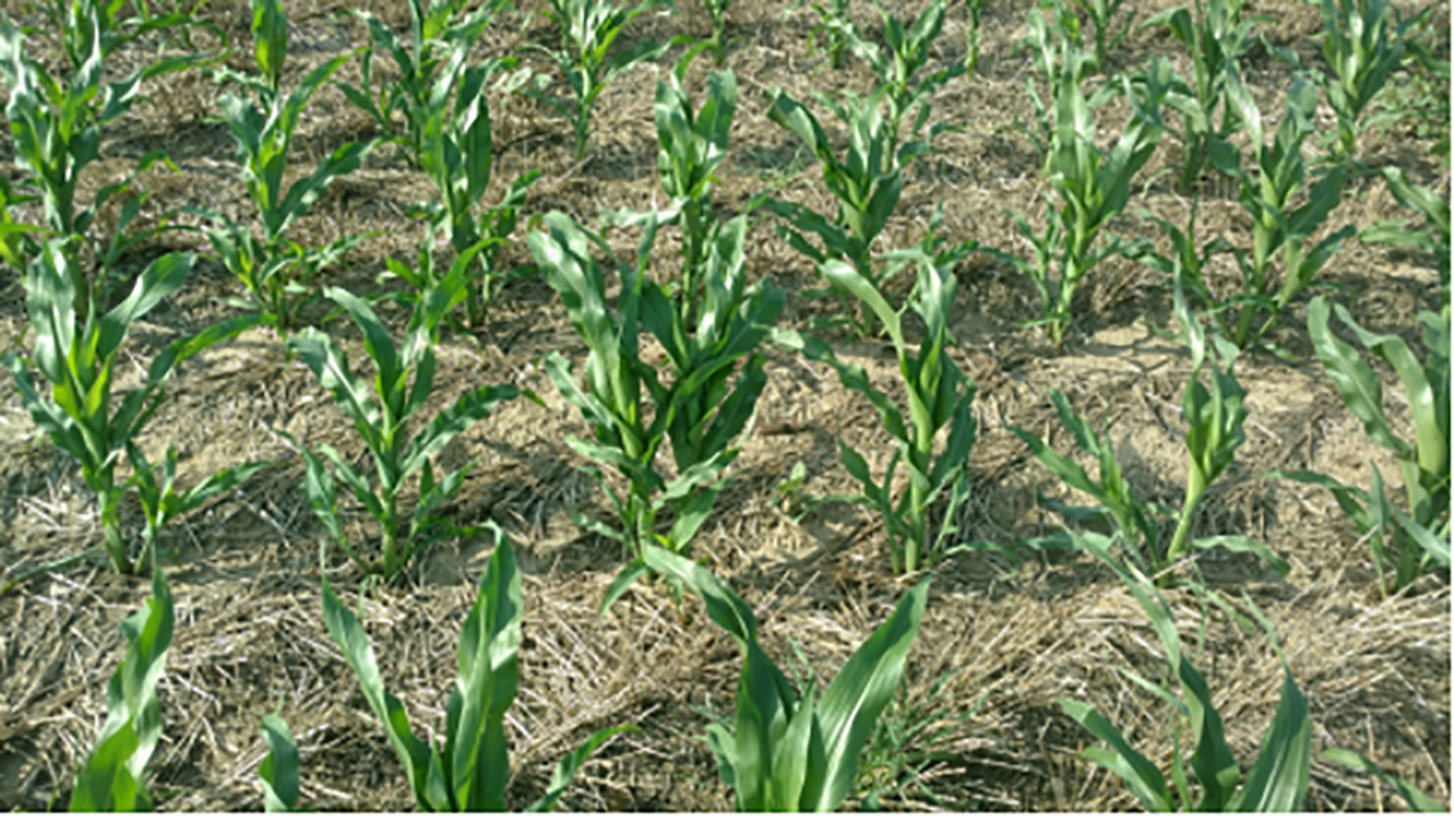
Corn planted in variable dryland environments (e.g., the U.S. High Plains and southwestern Argentinian Pampas regions) is often targeted at half the optimum plant density used by more productive counterparts (e.g., the U.S. Corn Belt). This mitigation practice adapts corn production to restrictive conditions and limited resources. However, tillering is more frequently observed at such low plant densities (< 50000 plants ha^-1^). The pictured field in Colby, Kansas, is in a wheat-corn-fallow crop rotation, and averages ~375 mm of precipitation during the corn growing season. Around the world, corn production is expanding into less conducive growing conditions. Understanding how to predict tiller development in these environments may facilitate creation of more holistic decision tools for producers seeking to maximize efficiency of corn crops.

Modern preferences of farmers and breeders alike commonly mask tiller capacity in corn fields, although this plasticity mechanism is conserved and situationally expressed (Moulia et al., 1999; Duvick et al., 2004). Common factors promoting tiller expression in corn are linked to plant density and are seasonal in nature (Downey, 1972; Markham and Stoltenberg, 2010). Interplant competition is minimized when plant density is reduced. Intentional plant density reductions are employed by farmers to match crop needs with limited resource availability, a pervasive component of dryland corn production illustrated by **Figure 1** (Roozeboom et al., 2007; Rotili et al., 2019). Unintentional plant density reductions include poor plant establishment or early season plant damage and death. In these situations, corn tillers may be expressed in row gaps or as a response to the released apical dominance of a damaged primary shoot (Carter, 1995; Thapa et al., 2018). Tillering in corn has historically lent itself to the theory that expression (“suckering”) reduced yields (Dungan, 1931; Earley et al., 1971). More recent studies have challenged this blanket opinion (Veenstra et al., 2021; Rotili et al., 2022).

While past work has quantified the impact of tillers on corn yield (Sangoi et al., 2009; Veenstra et al., 2021; Massigoge et al., 2022), plastic capacity (Rotili et al., 2021a; Rotili et al., 2022), and resource use (Thapa et al., 2018; Rotili et al., 2022), no substantial effort has been made to quantify the predictability of corn tiller presence in field scenarios. Corn yields were not reduced, but did respond to varying levels of tiller density (i.e., tillers ha^-1^) in previous work from the available database (Veenstra et al., 2021; Veenstra et al., 2023a). In addition, water-soluble carbohydrate reserves and plant C economy dynamics were altered by tiller presence (Veenstra et al., 2023b). Therefore, accurately predicting this plasticity behavior is relevant in the evaluation of corn tillering utility. Plasticity quantification remains a broad research gap, particularly in light of agronomic applications (Sadras et al., 2013). Such a gap provides opportunity to improve tools available to producers in marginal, variable, or otherwise vulnerable growing environments and enhance sustainability and resilience of these production systems. Environmental drivers were strongly correlated with yield responsiveness to tiller formation in Veenstra et al. (2021) and Massigoge et al. (2022) and similar variables have been proposed in a mechanistic framework for understanding this trait in corn (Rotili et al., 2021b). Therefore, it follows that such data should be useful in describing tiller densities at the field scale. Authors hypothesized that corn tiller densities in tiller-prone genotypes could be reliably predicted within 25% of the target plant density (arbitrarily identified as a realistic management threshold) using readily accessible variables related to crop management and environment. Thus, the objectives of this study were as follows: 1) identify key environment, management, or combinations of these variables useful for predicting tiller density dynamics in corn; and 2) test out-of-season prediction accuracy for identified variables.

## 2 Materials and methods

### 2.1 Field experiments

This study utilized data from 17 site-years of field experiments across the state of Kansas, United States in the 2019 to 2021 seasons, as previously described in Veenstra et al. (2021) and **Table 1**. At each site, treatments were applied in a split-split-plot arrangement with a randomized complete block design and replicated three or four times depending on the site-year, as field space allowed. Whole plot was assigned as plant density, with target levels 25000, 42000, and 60000 plants ha^-1^. These plant densities were selected as representative of low, moderate, and high plant density targets for the U.S. Central High Plains region of the United States (Roozeboom et al., 2007). Lower plant densities were used to simulate suboptimal stands in good environments and typical stands in restrictive dryland environments. Sub-plot was assigned as genotype, with levels P0805AM and P0657AM (Corteva Agriscience, Johnston, IA, USA). These genotypes were selected for their modern release date, suitability for the region and limited moisture production systems targeted, and propensity to tiller. As genetic influence was not the main focus of the study, two genotypes were deemed sufficient. Sub-sub-plot was assigned as tiller presence, with levels “intact” or “removed”. For the current study, only plots with undisturbed tillers were evaluated. Additional information on plot care and size can be found in the aforementioned article.

**Table 1:** Site-year field experiment coordinates, sow date, tenth-leaf (V10) date, treatment structure (D, Density; G, Genotype; P, Tiller Presence), irrigation, previous crop, and soil characterization [pH, organic matter (OM – loss on ignition), nitrate concentration (NO_3_-N), ammonium concentration (NH_4_-N), phosphorus (P – Mehlich), cation exchange capacity (CEC), and soil texture].

Site-Year	Latitude	Longitude	Sow Date	V10 Date	Treatment Structure	Irrigation	Previous Crop	pH	OM	NO_3_-N	NH_4_-N	P	CEC	Soil Texture
	(°N)	(°W)						(H_2_0)	% (LOI)	(mg kg^-1^)	(mg kg^-1^)	Mehlich (mg kg^-1^)	(meq 100g^-1^)	
Manhattan 2019	39.14	96.64	May-14	Jul-01	D x G x P	None	Corn	6.3	1.0	1.8	1.3	37.5	5.9	Sandy Loam
Garden City 2019	37.83	100.86	May-04	Jun-28	D x G x P*	Subsurface limited	Corn	6.6	1.0	2.0	0.0	42.0	15.8	Sandy Loam
Goodland 2019	39.25	101.78	May-14	Jul-08	D x G x P*	Subsurface limited	Soybean	6.5	2.7	26.8	2.1	52.1	18.2	Silt Loam
Keats 2020	39.23	96.72	May-02	Jun-24	D x G x P	None	Soybean	7.0	4.5	18.0	4.1	118.0	24.4	Silty Clay Loam
Buhler 2020	38.14	97.73	Apr-29	Jun-20	D x G	Subsurface limited	Soybean	6.4	2.9	17.9	4.8	24.0	23.1	Silty Clay Loam
Greensburg 2020	37.58	99.37	May-05	Jun-24	D x G	Subsurface limited	Corn	5.4	2.6	37.1	13.6	84.9	18.9	Clay Loam
Garden City 2020	37.83	100.86	May-18	Jun-30	D x G x P	Subsurface limited	Corn	5.2	1.6	18.4	10.7	55.0	10.6	Sandy Loam
Goodland 2020	39.25	101.78	May-07	Jul-01	D x G x P	Subsurface limited	Soybean	5.8	3.8	36.9	17.9	106.0	24.0	Silt Loam
Colby A 2020	39.39	101.06	May-07	Jul-03	D x G x P	None	Wheat	5.4	3.3	19.9	4.3	70.0	21.2	Silt Loam
Colby B 2020	39.38	101.06	May-15	Jul-03	D x G x P	None	Grain Sorghum	6.5	3.2	43.5	36.4	31.0	24.0	Silt Loam
Keats 2021	39.23	96.72	Apr-30	Jun-22	D x G x P	None	Corn	6.6	6.2	23.3	12.7	106.4	25.6	Silt Loam
Buhler 2021	38.14	97.73	May-04	Jun-25	D x G	Subsurface limited	Corn	6.3	2.6	11.7	7.8	13.3	22.3	Silt Loam
Greensburg 2021	37.58	99.37	May-07	Jun-25	D x G	Subsurface limited	Corn	5.6	2.3	33.4	7.4	68.8	20.0	Loam
Selkirk 2021	38.70	101.54	May-06	Jun-30	D x G	Subsurface limited	Field Bean	7.9	2.7	14.0	5.8	90.9	23.2	Loam
Garden City 2021	37.83	100.86	May-13	Jun-28	D x G x P	Subsurface limited	Corn	5.5	1.6	14.2	5.2	52.1	9.7	Sandy Loam
Goodland 2021	39.25	101.78	May-05	Jun-30	D x G x P	Subsurface limited	Soybean	6.5	2.9	36.9	11.1	65.4	23.2	Loam
Colby A 2021	39.39	101.06	Jun-04	Jul-15	D x G x P	None	Wheat	7.1	2.9	23.8	7.1	93.0	22.2	Clay Loam

The 2019 and 2020 sites are presented as described in Veenstra et al. (2021). *missing one level of plant density (D).

Morphology and phenology data were recorded in unique sections (at least 1.2 m^2^) of buffered central plot rows throughout the season in each study. Plant and tiller counts per area were taken at V5 (fifth leaf; Ritchie et al., 1997), V10 (tenth leaf), V16 (sixteenth leaf, 2019 and 2021), R3 (kernel milk stage, 2019 and 2021), and R6. Crop phenology was noted at each collection to ensure timing was on-target. Counts were scaled to plants ha^-1^ and tillers ha^-1^.

### 2.2 Environmental data and calculations

No soil fertility or moisture gradients (ex: irrigated versus dryland) were established at the evaluated locations. Only field-level soil and weather data were collected and evaluated. Soil type and fertility were characterized for each site-year via early-season soil sampling at 60-cm (NO_3_ and NH_4_) and 15-cm depths (all others, **Table 1**). Weather data were obtained from the Climate Engine web application for all desired geographic locations, using coordinates and date ranges (Huntington et al., 2017). Soil bulk density data were downloaded from the Web Soil Survey application (Staff S.S., Service N.R.C. and Agriculture, U.S.D, 2022) and used to calculate nutrient values in kg ha^-1^. In total, 16 environmental variables were available for study, which were grouped into 15 categories based on previous knowledge of importance to tiller response (Veenstra et al., 2021), ease of producer manipulation (i.e., management factors – including plant density, amendable soil variables, and water as irrigation), and field observations. All calculations, data transformation, and analyses were conducted using program R (R Core Team, 2022).

All climate data considered the time period from planting to date of observation, regardless of plant development stage. While previous work has indicated critical periods for tiller appearance (Moulia et al., 1999; Rotili et al., 2021b), authors wished to capture seasonal trends that could impact tiller density through abortion as well. Mechanistic relationships are not well-defined in this regard (e.g., tiller abortion has been related to phenological progression, but this could be more influenced by seasonal stress or soil water depletion than plant development alone). Cumulative values (additive from planting to field observation date) included growing degree days (GDD), vapor pressure deficit (VPD), and soil water supply. Daily GDD were calculated as the difference between mean daily temperature and the crop base temperature of 10°C, with a forced daily maximum of 30°C. The VPD was included as a proxy of crop stress in addition to temperature, provided in kilopascals (kPa), and added over the specified date range. Soil water supply included both precipitation and irrigation, when applicable, with the season extended one month pre-sowing to estimate soil moisture at planting. Daily minimum temperature, daily maximum temperature, daily thermal amplitude, and photothermal quotient (PTQ) were averaged over the assigned period (planting to field observation). The PTQ is defined by Fischer (1985) as the mean daily solar radiation for a selected time period, divided by the difference between the mean temperature for that period and the crop base temperature (10°C), with final units megajoules (MJ) m^-2°^C^-1^ day^-1^.

### 2.3 Data analyses

#### 2.3.1 Model specification, fit, and selection

**Figure 2:**
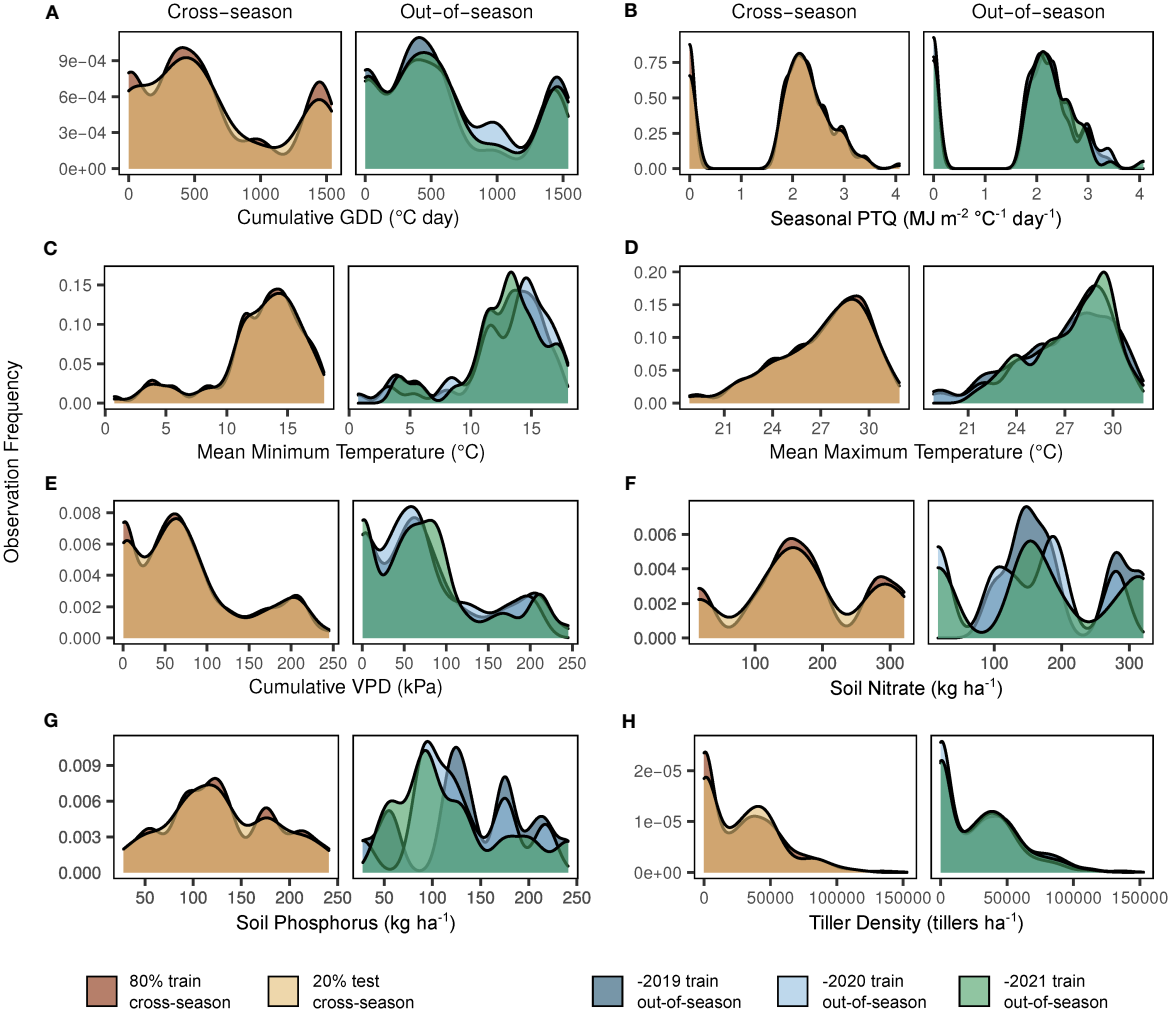
Observation frequency for key factors in dataset model splits. Y-axes indicate the observation frequency for each factor within a given dataset split. Factors shown are based on the selected model structure (**Supplementary Equation S1**) and include cumulative growing degree days (GDD; **A**), seasonal photothermal quotient (PTQ; **B**), mean minimum and maximum temperatures **(C, D)**, cumulative vapor pressure deficit (VPD; **E**), soil nitrate **(F)**, soil phosphorus **(G)**, and response variable tiller density **(H)**. The left side of each panel demonstrates the cross-season 80% train (dark), 20% test (light) dataset split. The right side of each panel demonstrates the seasonal variation of the out-of-season (-2019, dark; -2020, pale; -2021, moderate) dataset splits.

In preparation for model fitting and evaluation, the complete dataset (multiple sites, seasons, and development stages) was divided into training and testing sets – 80% and 20% (4:1), respectively. This split was selected as a compromise between maximizing the training dataset and maintaining the integrity of both training and testing sets (Gholamy et al., 2018). These initial sets had similar representation of all site-years, potential predictor variables, and observed tiller densities (**Figure 2**). The training set (80%) was utilized for model fitting (e.g., estimation of regression coefficients). The testing set (20%) was sacrificed for model testing only and reserved to evaluate the predictive accuracy and adequacy of the model fit to the training set. This 4:1 model is henceforth referred to as the “cross-season” fit.

The 15 different variable combinations selected as potential predictors of tiller density are presented in **Table 2**. To facilitate useful interpretation of interactions among non-categorical variables, observed plant density was categorized into three clusters based on target plant densities of 25000, 42000, and 60000 plants ha^-1^, of which realized densities were representative (data not shown). These factor levels were utilized when plant density was involved in interactions with at least one other continuous variable. As generalized additive models require a distribution representative of the response variable, we selected a Binomial distribution for realization of tillers ha^-1^. Essentially, this assumption allowed us to determine the probability (ranging from 0 to 1) of attaining a maximum potential tiller density ha^-1^ as the response variable. The assumed maximum achievable tiller expression at a field scale was based on findings of others (3 tillers plant^-1^; Major, 1977; Rotili et al., 2021a), and a realistic maximized plant density of 100000 plants ha^-1^, as utilized in previous plant density studies in the U.S. (Assefa et al., 2016). The potential tiller density in our model was therefore 0 ≤ *y* ≤ 300000 tillers ha^-1^, where *y* is the predicted tiller density expressed as *m* × 300000 tillers ha^-1^, with *m* being the modeled probability of attaining a maximized tiller density per area. Such a tiller density has never been reported in the literature and is arguably not achievable. The highest observed tiller density in this study was 152842 tillers ha^-1^ (mean of 0.8 tillers plant^-1^, with 2% of individual observations > 3 tillers plant^-1^). The mean plant density in the current study was 41295 plants ha^-1^, ranging from 17514 to 73807. A maximum tiller density at the field level determined independently of present observations minimizes assumptions of tiller responses to plant density, for example. Such responses have been reported at the tillers plant^-1^ scale (Downey, 1972; Tetio-Kagho and Gardner, 1988; Sangoi et al., 2009; Hansey and de Leon, 2011) but authors believe tiller density drivers at the field scale (tillers ha^-1^) are too uncertain (Downey, 1972) to necessitate constraints based on specific variables. All generalized additive models were fit to the training set using the *mgcv* package with all continuous variables set as flexible smoothed effects via thin-plate regression splines (Wood, 2003). Limiting thresholds for each of the selected variables were defined based on a 0.50 probability.

**Table 2:** Predictive factors included in tiller density model candidates.

Model Candidate	GDD	PTQ	T_min_	T_max_	T_amp_	CM	VPD	PD	G	pH	OM	NO_3_	NH_4_	P	CEC	Sand	Silt	Clay
	°C day	MJ m^−2°^C^−1^ day^−1^	°C	°C	°C	mm	kPa	plants ha^-1^			% (LOI)	(kg ha^-1^)	(kg ha^-1^)	(kg ha^-1^)	(meq 100g^-1^)	%	%	%
Full	✓	✓	✓	✓	✓	✓	✓	✓	✓	✓	✓	✓	✓	✓	✓	✓	✓	✓
Temporal	✓	✓																
Weather	✓	✓	✓	✓	✓	✓	✓											
Soil										✓	✓	✓	✓	✓	✓	✓	✓	✓
E	✓		✓	✓	✓	✓	✓			✓	✓	✓	✓	✓	✓	✓	✓	✓
M	✓					✓		✓	✓	✓		✓	✓	✓				
Stress			✓	✓		✓	✓											
G + E	✓	✓	✓	✓			✓		✓			✓		✓				
G × E	✓	✓	✓	✓			✓		✓			✓		✓				
E + M	✓	✓	✓	✓			✓	✓				✓		✓				
E × M	✓	✓	✓	✓			✓	✓*				✓		✓				
G + M								✓	✓									
G × M								✓	✓									
G + E + M	✓	✓	✓	✓			✓	✓	✓			✓		✓				
G × E × M	✓	✓	✓	✓			✓	✓*	✓			✓		✓				

G, genotype; E, environment; M, management; GDD, cumulative growing degree days; PTQ, growing period photothermal quotient; T_min_, mean daily minimum growing period temperature; T_max_, mean daily maximum growing period temperature; T_amp_, mean daily growing period thermal amplitude; CM, cumulative seasonal moisture (precipitation + irrigation, when applicable); VPD, cumulative vapor pressure deficit; PD, observed plant density; pH, soil test pH; OM, soil test organic matter (percent loss on ignition); NO_3_, soil nitrate; NH_4_, soil ammonium; P, soil phosphorus; CEC, soil test cation exchange capacity; Sand, percent soil sand; Silt, percent soil silt; Clay, percent soil clay. * PD classified into three factor levels (A, 25000 plants ha^-1^; B, 42000; C, 60000).

Predictive accuracy was evaluated by the mean absolute error and mean bias error of out-of-sample predictions in the test set. Smaller values of mean absolute error indicate increased predictive accuracy. The flexible smooth effects (i.e., probability of achieving maximum tiller densities) for predictor variables in the most accurate model were independently plotted across the range of corresponding observations.

#### 2.3.2 Out-of-season evaluation

After the most accurate predictive model was identified for the cross-season dataset, the same model structure was evaluated for out-of-season predictive accuracy. The goal of such tests was to determine strengths and weaknesses of the predictive model for in-field agronomic applications in a new, untrained season. To test the predictive accuracy of the model for one year (2019, for example), the training dataset included only observations from the other two years (in this case, 2020 and 2021). The splits for these sets (percent train/percent test) were 80/20, 62/38, and 58/42 for 2019, 2020, and 2021, respectively. These fits are henceforth referred to as “out-of-season” models.

To evaluate out-of-season model performance, predictive accuracy for each site-year was determined as the difference between the true tiller density and the corresponding point prediction at the mean target plant density of 42000 plants ha^-1^ (representative of the true observed mean, 41295 plants ha^-1^). To explore predictive error causation in each site-year, out-of-season coefficient estimates were independently set to zero. Resulting mean absolute error was calculated for each exclusion. The excluded coefficient that most drastically reduced mean absolute error for a given site-year was identified as the variable too heavily weighted for the observed conditions in a given out-of-season fit.

## 3 Results

### 3.1 Model training and selection

Observation distributions in model training/testing splits for cross-season and out-of-season datasets are shown in **Figure 2**. Observations were relatively similar for all sets with regard to cumulative growing degree days (**Figure 2A**), seasonal photothermal quotient (**Figure 2B**), mean minimum and maximum temperatures (**Figures 2C, D**), cumulative vapor pressure deficit (**Figure 2E**), and tiller density (**Figure 2H**). Although NO_3_ (**Figure 2F**) and P (**Figure 2G**) observations were similar between the cross-season sets, as expected, seasonal variation was evident in the out-of-season sets.

The mean observed tiller density in the full dataset was 28781 tillers ha^-1^ and the mean observed plant density was 41295 plants ha^-1^. Therefore, an acceptable error range across the dataset (< 25% of the plant density) was < 10324 tillers ha^-1^. This error level was selected as an arbitrarily significant margin that farmers could meaningfully use to inform management practices. Considering the 15 variable combinations, out-of-sample prediction mean absolute error is presented for each model fit in **Table 3**. Models including weather-based factors were consistently below the targeted error threshold. The three most accurate models were the simplified E × M (mean absolute error = 7776 tillers ha^-1^; mean bias error = -38 tillers ha^-1^), the simplified G + E + M (mean absolute error = 9050 tillers ha^-1^), and the full model (mean absolute error = 9051 tillers ha^-1^). The simplified E × M model was selected as the most appropriate (**Supplementary Equation S1**). Plant density was the key management factor identified, and the most relevant environmental factors were temperature- (GDD, PTQ, VPD, mean minimum and maximum daily temperatures) and soil fertility-related (NO_3_ and P).

**Table 3:** Prediction accuracy metrics for tiller density model candidates.

Model Candidate	MAE (tillers ha^-1^)
Full	9051
Temporal	13908
Weather	9362
Soil	19560
E	9362
M	11377
Stress	10605
G + E *	9116
G × E *	9736
E + M *	9066
E × M *	7776
G + M *	22764
G × M *	23131
G + E + M *	9050
G × E × M *	10371

Lowest values for each distribution are shown in boldface type. MAE, mean absolute error of cross-season, out-of-sample predictions for test data set (80% train, 20% test); E, environment; M, management; G, genotype. *simplified to key parameters (selected to minimize MAE; see **Table 1**).

### 3.2 Independent predictor effects

**Figure 3:**
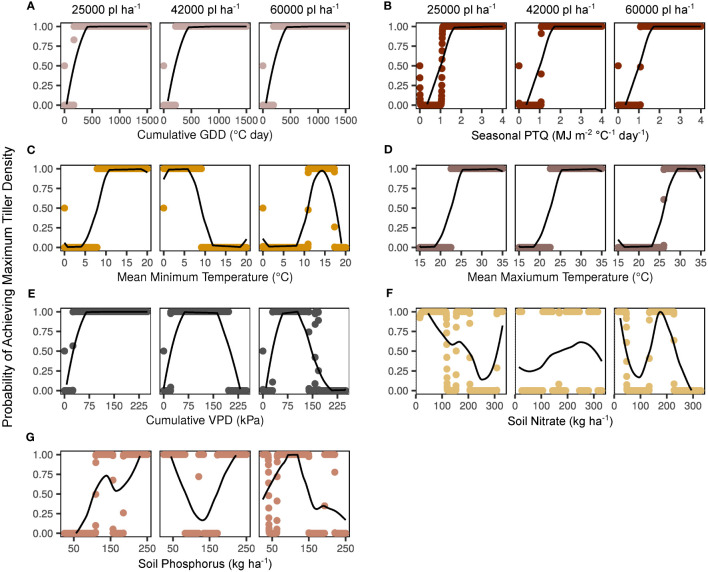
Independent smooth functions fitted to key environmental factors by target plant density. Y-axes indicate the model-generated probability of a maximum tiller density observation (300000 tillers ha^-1^) across a range of potential factor levels. Probabilities for each variable level are shown with points, and moving averages are indicated with lines. Factors shown include cumulative growing degree days (GDD; **A**), seasonal photothermal quotient (PTQ; **B**), mean minimum and maximum temperatures **(C, D)**, cumulative vapor pressure deficit (VPD; **E**), soil nitrate **(F)**, and soil phosphorus **(G)**.

Smoothed effects of predictor variables on maximum tiller density probability are presented independently in **Figure 3**. It is key to note that the greatest tiller density observed in the present study was 152842 tillers ha^-1^. That is, even when a variable was apparently non-limiting, other limiting variables prevented the observed density from reaching the set theoretical maximum of 300000 tillers ha^-1^. Cumulative GDD had a consistent impact on tiller probabilities across plant densities, with a clear threshold of > 200°C day identified as non-limiting (**Figure 3A**). The effect of seasonal PTQ was steady across plant densities, with a non-limiting threshold of > 1 MJ m^-2^ C^-1^ day^-1^ (**Figure 3B**). Non-limiting mean minimum temperature thresholds were > 8°C for the 25000 plants ha^-1^ target, < 9°C for 42000 plants ha^-1^, and < 17.5 but > 11°C for 60000 plants ha^-1^ (**Figure 3C**). Mean maximum temperature was consistently non-limiting > 22°C for 25000 and 42000 pl ha^-1^ and > 26°C for 60000 pl ha^-1^ (**Figure 3D**). Cumulative VPD was limiting below 20-30 kPa for all plant densities, but was also limiting to some degree at higher values for the 42000 (200 kPa) and 60000 (150 kPa) plants ha^-1^ targets (**Figure 3E**). Soil fertility impacts were less consistent overall (**Figures 3F, G**). At 25000 pl ha^-1^, increasing kg NO_3_ ha^-1^ appeared to have detrimental impacts on tiller expression beyond 200 kg ha^-1^, whereas P had a clearer minimum threshold of 100 kg ha^-1^. Tiller response was fairly stable across nutrient gradients for both NO_3_ and P at 42000 pl ha^-1^. Highest values of NO_3_ and P were the most limiting at 60000 pl ha^-1^, with the apparent NO_3_ threshold at 230 kg ha^-1^ and the P threshold at 150 kg ha^-1^. These independently presented variables are correlated with each other, with the most correlated associations (r > 0.7) as cumulative vapor pressure deficit with cumulative growing degree days, mean maximum with mean minimum temperature, mean maximum temperature with cumulative vapor pressure deficit, and mean temperatures with cumulative growing degree days (data not shown).

### 3.3 Out-of-season predictive accuracy

**Figure 4:**
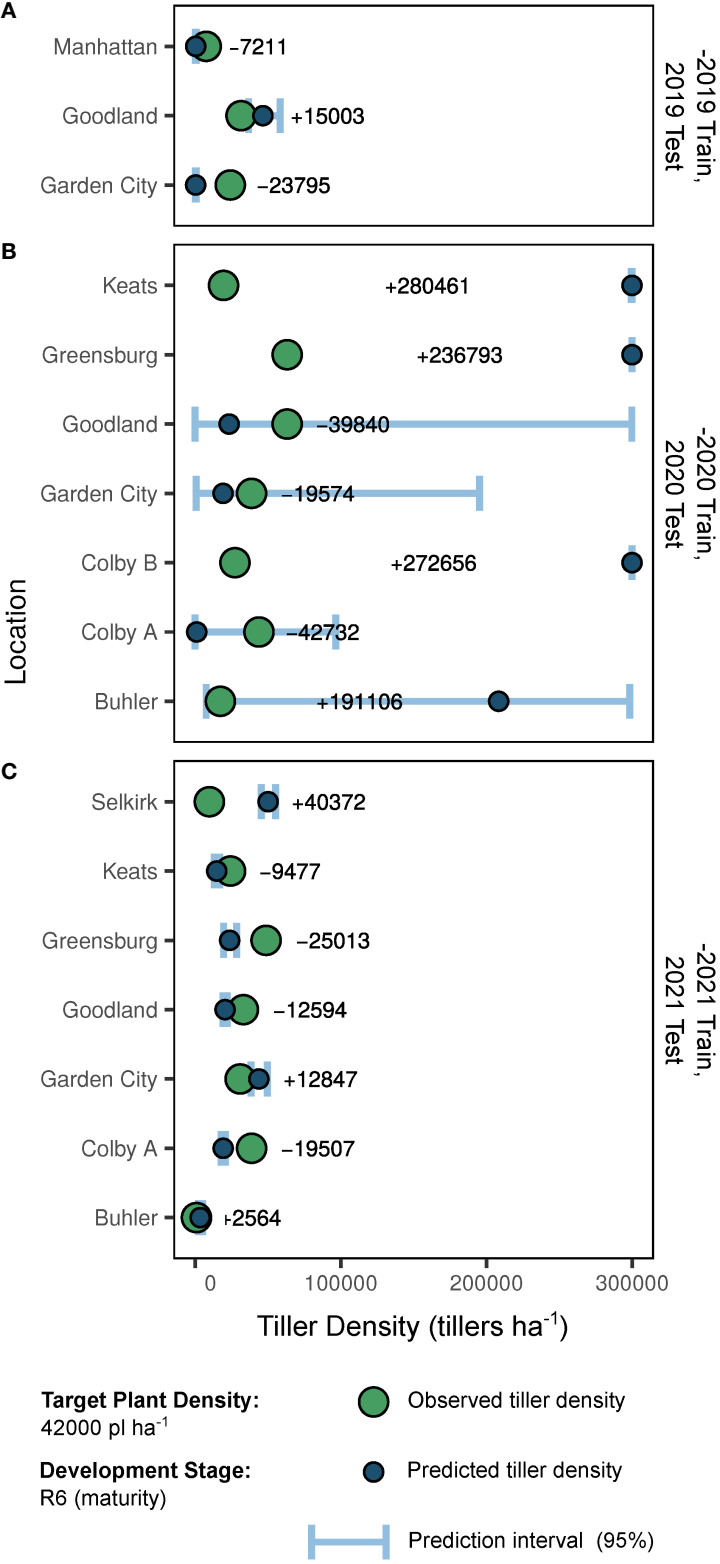
Predictions and predictive accuracy of out-of-season model fits (-2019, **A**; -2020, **B**; -2021, **C**) by site-year. As presented, “-2019” indicates the model trained with 2020 and 2021 data but tested with 2019 data. Large points indicate true tiller density observations for a plant density of 42000 plants ha^-1^ at development stage R6 (physiological maturity). Small points indicate model-generated predictions for tiller density (also 42000 plants ha^-1^ at stage R6). Error bars indicate the 0.95 quantile prediction interval. In-row text indicates the error of the point prediction compared to the observed value for a given site-year.

Out-of-season predictions and resulting accuracy are presented in **Figure 4**. Overall, predictions for the -2021 and -2019 trained models were most accurate. When calculated via the -2019 coefficient estimates, 2019 tiller density predictions ranged in absolute error from 7211 to 23795 tillers ha^-1^, and averaged -16003 tillers ha^-1^ (**Figure 4A**). When calculated via the -2020 coefficient estimates, 2020 tiller density predictions ranged in absolute error from 19574 to 280461 tillers ha^-1^, and averaged +125553 tillers ha^-1^ (**Figure 4B**). When calculated via the -2021 coefficient estimates, 2021 tiller density predictions ranged in absolute error from 2564 to 40372 tillers ha^-1^, and averaged -1544 tillers ha^-1^ (**Figure 4C**).

### 3.4 Out-of-season error evaluation

Prediction accuracy impacts of independent out-of-season coefficient estimate removal are presented in **Table 4**. When select out-of-season coefficient estimates were excluded, 59% of site-years dropped below the reasonable error threshold of 10500 tillers ha^-1^ (25% of 42000 plants ha^-1^ target). The 2020 predictions were responsible for 71% of the unacceptable error. Coefficients most improperly weighted for 2019 sites were soil NO_3_ and mean maximum temperatures. Coefficients most improperly weighted for 2020 sites were growing degree days, soil P, soil NO_3_, and photothermal quotient. The site-years most over-predicted by the full model in 2020 had the highest and lowest observed NO_3_ and P kg ha^-1^ in the collected dataset. Predictions for two 2020 sites were most accurate with all coefficient estimates. Coefficients most improperly weighted for 2021 sites were temporal and temperature-related (vapor pressure deficit, growing degree days, minimum temperature, and maximum temperature).

**Table 4:** Out-of-season error evaluation resulting from independent coefficient eliminations by site-year.

Model	Location	Full Error	Lowest Error	Zeroed Coefficient
-2019 Train, 2019 Test	Manhattan	7211	4099	VPD
	Goodland	15003	5963	NO_3_
	Garden City	23795	13366	T_min_
-2020 Train, 2020 Test	Keats	280461	10283	P
	Greensburg	236793	56352	NO_3_
	Goodland	39840	39840	
	Garden City	19574	19574	
	Colby B	272656	27344	PTQ
	Colby A	42732	27631	P
	Buhler	191106	1653	P
-2021 Train, 2021 Test	Selkirk	40372	9770	VPD
	Keats	9477	7280	GDD
	Greensburg	25012	11851	T_min_
	Goodland	12594	6547	GDD
	Garden City	12847	1058	T_max_
	Colby A	19507	8579	GDD
	Buhler	2564	912	VPD

VPD, cumulative vapor pressure deficit; GDD, cumulative growing degree days; PTQ, growing period photothermal quotient; T_max_, mean daily maximum growing period temperature; T_min_, mean daily minimum growing period temperature; NO_3_, soil nitrate; P, soil phosphorus.

## 4 Discussion

From a diverse dataset of field experiments, this study presents novel conclusions on the predictability of tiller densities in selected modern corn genotypes. While recently published literature has explored the general yield and reproductive outcomes of corn tiller presence in modern farm management systems (Rotili et al., 2021a; Veenstra et al., 2021; Massigoge et al., 2022; Rotili et al., 2022; Veenstra et al., 2023a; Veenstra et al., 2023b), no substantial effort has been made to explore appearance and survival factors for corn tiller densities. In an effort to fill this knowledge gap, G, E, and M variables were evaluated in replicated, multi-season, state-wide field trials in Kansas, U.S. These 17 site-years comprise an expansive tiller-focused database which has offered numerous yield and reproductive plasticity insights for corn management in the US Central High Plains region (Veenstra et al., 2021). The expanse of this dataset facilitates unique modelling approaches (e.g., GAMs) not typically possible in traditional field experiments evaluated *via* ANOVA and mixed models. The current study provides perspective of tiller density drivers and out-of-season tiller density predictability for selected corn genotypes in a range of environments, management practices, and crop stages.

A key component for selected predictor variables is the Sprengel-Liebig Law of the Minimum (van der Ploeg et al., 1999). This foundation discloses that none of the insignificant parameters were obviously limiting in any of the evaluated trials. If this assumption is not met, model predictive accuracy may be degraded. Moisture supply was not a significant factor, likely because no evaluated environments were critically water-restricted. Previous simulation work has indicated tiller productivity response to water supply (Rotili et al., 2021a), although presented data suggest water supply may be a factor indirectly controlling tiller expression (via reduced photosynthetic capacity, for example). Diverse, field-based data sets for model training are imperative, as evidenced by the out-of-season predictions and resulting error margins in the current study. Maximum temperature, temporal, and fertility coefficients were most commonly overweighed in the out-of-season training data sets, solidifying the observation that these factors are better classified as benchmark indicators (e.g., minimum or maximum for expression) than as tiller density drivers. Studies by Rodriguez et al. (1999) indicated that P deficiency altered the wheat phyllochron and subsequently reduced tiller emergence rate. In addition, a greater diversity of genotypes could uncover alternate tiller expression responses, as supported by previous work (Hansey and de Leon, 2011).

Two main hypotheses are generally ascribed to tiller development in corn – 1) tillering is regulated by red:far-red ratios alone and/or 2) tillering is regulated by red:far-red ratios and energy (sugars). Identified by previous work for corn and other crop species, season timing, the PTQ, and thermal variables were crucial predictive components for tiller densities. Corn tiller initiation was found to follow the typical grass species delay of one phyllochron from main shoot development (Moulia et al., 1999) and has been previously described with thermal time (Rotili et al., 2021b). Rotili et al. (2021b) presented data from Maddonni et al. (2002), which began measurements at 500°C days. Tillers plant^-1^ were as high as 0.5 for some initial values, indicating the limiting value was < 500°C days. The red to far-red light ratio was also key to tiller development in this study by Maddonni et al. (2002). Kim et al. (2010b) indicated that grain sorghum tiller appearance began between 150 and 250°C days and PTQ was a useful indicator of tillering potential. These observations correspond with the threshold of 200°C days identified here for corn, as well as the importance of PTQ as a potentially limiting factor in tiller expression. Related to this point, C economy is commonly equated with tiller expression. However, C depletion was not associated with reduced tiller densities in grain sorghum studies by Lafarge and Hammer (2002). Plant density is likely the indirect cause, as red:far red light quality is impacted by a fuller canopy (Markham and Stoltenberg, 2010).

Plant density was the key management factor significantly altering the outcome of tiller densities in the current study, as previously reported for corn at the plant scale (Major, 1977; Tetio-Kagho and Gardner, 1988; Hansey and de Leon, 2011; Rotili et al., 2021b) and field scale (Downey, 1972). The factors most clearly interacting with plant density in this study were VPD and maximum temperature. Thorne and Wood (1987) observed reduced tiller number with high temperatures in pre-kernel set periods of wheat (a C3 species). In addition, greater radiation encouraged tiller development, although onset of heat treatments canceled out this effect at harvest (Thorne and Wood, 1987). The current study identified a base threshold for high temperature rather than an upper threshold (potentially attributed to the C4 nature of corn) and a base threshold for low temperatures. In contrast to minimum temperatures, the increasing threshold for maximum temperatures is consistent with the photosynthetic response to temperature. For example, tiller expression can be maximized at sub-optimal growing temperatures with low plant densities, but greater temperatures, maximizing photosynthetic rates, are required to observe tillers in higher plant densities. Cumulative VPD was associated with tiller expression as a base threshold, likely due to the temporal nature of how this variable was calculated. Considered as a stress index for this study, however, high cumulative VPD captured tiller abortion responses not attributed to growing degree days alone at 42000 and 60000 plants ha^-1^ densities. Growth rates, integral to tillering responses in corn (Andrade et al., 1999; Kamiji et al., 2011; Rotili et al., 2022), are negatively impacted by high VPDs. Therefore, a lower ceiling in higher plant densities is required to maintain growth per plant. The VPD is associated with heterogeneity in corn phenology and yield, which could facilitate plasticity in certain environments (Lobell and Azzari, 2017). Additional work should be done to partial out plant growth rate impacts on tiller expression, development, and dynamics of these to explore physiological processes and improve crop models in this regard.

Soil fertility, specifically NO_3_ and P, were significant to predictive accuracy, as expected based on precedent in wheat (Thorne and Wood, 1987; Rodriguez et al., 1999), rice (Alam et al., 2009), and grain sorghum (van Oosterom et al., 2010). Adequate P levels are crucial to hormonal branching responses in plants, mitigating the apical dominance conferred by strigalactones and promoting production of cytokinins (Yan et al., 2020). Relationships and thresholds for soil fertility variables in tiller expression probability were less apparent than for weather factors, but these parameters are certainly important. Continued corn tiller field studies should include fertility as a dosed treatment factor to evaluate this response more formally with a factorial design approach.

Although weather factors appear to be reliable tiller density predictors, the utility of models dependent on future observations is an important caveat. However, out-of-season data forecasting (i.e., weather) is the common scapegoat for prediction challenges. This study clearly demonstrates the power of predictive distribution uncertainty, as out-of-season prediction intervals for some site-years were quite wide. Even if “reliable” data is available (i.e., observed in-season weather and soil data), uncertainty remains high in our attempt to replicate reality in biological systems. A diverse dataset is required to properly train such prediction models. This is demonstrated by the error of an appropriately trained model (**Figure 4C**) and the error reduction following removal of certain coefficients (**Table 4**). In some cases, such as the Goodland and Garden City sites in 2020, the growing season may not have been well-represented by the training dataset. Prediction error was not impacted by removal of a single coefficient, and prediction intervals were quite wide. In both 2019 and 2021 seasons however, our error levels were quite reasonable. That is, prediction of tiller expression at the field scale with limited in-field measurements (and identification of critical drivers) is possible, although some uncertainty in prediction of biological responses will inevitably remain.

The key challenge moving forward in such studies is making useful recommendations for stakeholders given the model-limited clarity (Gneiting and Katzfuss, 2014; Raftery, 2016). Introducing risk-reward perspectives of economics, environment, etc. may aid in generating such actionable decisions, but such ideals are difficult to quantify and vary by individual (Williams and Hooten, 2016; Mase et al., 2017; Komarek et al., 2020). These model predictions are likely not useful to assist farmers with plant density selection based on tiller expression for an upcoming season. However, in replant situations, increased certainty with season progression may generate an actionable prediction of tiller yield compensation potential. Outcomes of this study are useful for in-season diagnostics and concerns of year-to-year variation in tiller densities. Identifying predictors and consequences of crop plasticity improves understanding of how overlooked traits may enhance the resilience of our agroecosystems (Sadras et al., 2013). This is accomplished, in part, by providing clarity for resource use and crop potential in dynamic growing climates (from both adverse and favorable perspectives). While tillering has been identified as a potential source of useful plasticity for corn, knowledge of factors contributing to tiller productivity remains limited. Kernels from ears on tillers are key to yield compensation of tillered corn phenotypes (Massigoge et al., 2022; Rotili et al., 2022), but tiller reproductive development is not well understood. Not all tillers may equally contribute to plant productivity (Schaffner, 1930; Bonnett, 1948; Alofe and Schrader, 1975; Russelle et al., 1984).

The hypothesis put forth for this study *“that tiller densities can be reasonably predicted (i.e., within 25% of the target plant density) via environmental factors”* appears to be supported by our results. Plant density, thermal parameters, and soil fertility were critical components to achieving the lowest error in tiller density prediction. The cross-season predictive accuracy of identified models fell within the reasonable benchmark of < 25% observed plants ha^-1^, although not all out-of-season fits performed equally. Critical non-limiting thresholds for select environmental parameters were apparent in coefficient estimates. Wide prediction intervals highlighted the volatile nature of tiller expression and model assumptions, but point predictions were relatively good with sufficiently diverse training data. While useful for early season diagnostic purposes, these models are limited in forecast utility and should be coupled with appropriate decision theory and risk assessments. Future studies should expand on tiller density prediction by exploring how those tillers develop through the season.

## Data availability statement

The raw data supporting the conclusions of this article will be made available by the authors, without undue reservation.

## Author contributions

Conceptualization: RV, CM, PP, and IC. Methodology: RV, TH, and IC. Formal Analysis: RV and TH. Investigation: RV. Resources: LH and IC. Writing – original draft preparation: RV. Writing – review and editing: RV, TH, CM, LH, PP, and IC. Visualization: RV. Supervision: IC. Project Administration: RV and IC. Funding Acquisition: IC. All authors contributed to the article and approved the submitted version.
