# Supplementary material for: Predicting corn tiller development in restrictive environments can be achieved to enhance defensive management decision tools for producers
Source: Front Plant Sci. 2023 Aug 3;14:1223961. doi: 10.3389/fpls.2023.1223961 (PMC10436094; doi:10.3389/fpls.2023.1223961)
Supplement: Supplementary file 1 [file DataSheet_1.pdf]

Supplementary Material

Equation S1. Selected model for prediction of tiller densities in corn fields of various target plant densities and nutrient management strategies, considering non-limiting moisture availability and sufficiency of other potential limiting factors not listed.

- Data model: $z_i = y_i$ or $z_i \propto y_i$.
- Process model: $[y_i|\alpha_i] \equiv$ Binomial(α_i), where α_i = inverse logit$[\beta_0+f_{1d}(g_{id})+f_{2d}(q_{id})+f_{3d}(l_{id})+f_{4d}(x_{id})+f_{5d}(v_{id})+f_{6d}(n_{id})+f_{7d}(p_{id})]$.
- Parameter model: $\beta_0 \propto 1$.

That is,

- z_i is the observed tiller density ha^{-1}, assumed to be the same as y_i, the true density;
- α_i is the bulk of the process model as described above;
- β_0 is the y-intercept of fitted model;
- d is a plant density cluster with factor levels A (25000 plants ha^{-1}), B (42000 plants ha^{-1}), or C (60000 plants ha^{-1});
- f_{1d} is the smooth effect of cumulative growing degree days (GDD) for plant density cluster d;
- g_{id} is the cumulative GDD of observation i in plant density cluster d;
- f_{2d} is the smooth effect of photothermal quotient (PTQ) for plant density cluster d;
- q_{id} is the PTQ of observation i in plant density cluster d;
- f_{3d} is the smooth effect of mean minimum temperature for plant density cluster d;
- l_{id} is the mean seasonal minimum temperature of observation i in plant density cluster d;
- f_{4d} is the smooth effect of mean maximum temperature for plant density cluster d;
- x_{id} is the mean seasonal maximum temperature of observation i in plant density cluster d;
- f_{5d} is the smooth effect of the cumulative vapor pressure deficit (VPD) for plant density cluster d;
- v_{id} is the cumulative VPD of observation i in plant density cluster d;
- f_{6d} is the smooth effect of soil test nitrate (NO_3) for plant density cluster d;
- n_{id} is the soil test NO_3 (kg ha^{-1}) of observation i in plant density cluster d;
- f_{7d} is the smooth effect of soil test phosphorus (P) for plant density cluster d; and
- p_{id} is the soil test P (kg ha^{-1}) of observation i in plant density cluster d.
